# Physiological and Morphological Aspects of *Aedes aegypti* Developing Larvae: Effects of the Chitin Synthesis Inhibitor Novaluron

**DOI:** 10.1371/journal.pone.0030363

**Published:** 2012-01-24

**Authors:** Luana C. Farnesi, José M. Brito, Jutta G. Linss, Marcelo Pelajo-Machado, Denise Valle, Gustavo L. Rezende

**Affiliations:** 1 Instituto Oswaldo Cruz, Fiocruz, Rio de Janeiro, Rio de Janeiro, Brazil; 2 Instituto de Biologia do Exército, Rio de Janeiro, Rio de Janeiro, Brazil; 3 Instituto Nacional de Ciência e Tecnologia em Entomologia Molecular, Rio de Janeiro, Rio de Janeiro, Brazil; 4 Instituto de Ciências Biomédicas, Universidade Federal do Rio de Janeiro, Rio de Janeiro, Rio de Janeiro, Brazil; 5 Centro de Biociências e Biotecnologia, Universidade Estadual do Norte Fluminense Darcy Ribeiro, Campos dos Goytacazes, Rio de Janeiro, Rio de Janeiro, Brazil; University of Otago, New Zealand

## Abstract

Population control of the dengue vector mosquito, *Aedes aegypti*, is difficult due to many reasons, one being the development of resistance to neurotoxic insecticides employed. The biosynthesis of chitin, a major constituent of insect cuticle, is a novel target for population control. Novaluron is a benzoylphenylurea (BPU) that acts as a chitin synthesis inhibitor, already used against mosquitoes. However, information regarding BPU effects on immature mosquito stages and physiological parameters related with mosquito larval development are scarce. A set of physiological parameters were recorded in control developing larvae and novaluron was administered continuously to *Ae. aegypti* larvae, since early third instar. Larval instar period duration was recorded from third instar until pupation. Chitin content was measured during third and fourth instars. Fourth instars were processed histochemically at the mesothorax region, stained with hematoxylin and eosin (HE) for assessment of internal tissues, and labeled with WGA-FITC to reveal chitinized structures. In control larvae: i) there is a chitin content increase during both third and fourth instars where late third instars contain more chitin than early fourth instars; ii) thoracic organs and a continuous cuticle, closely associated with the underlying epidermis were observed; iii) chitin was continuously present throughout integument cuticle. Novaluron treatment inhibited adult emergence, induced immature mortality, altered adult sex ratio and caused delay in larval development. Moreover, novaluron: i) significantly affected chitin content during larval development; ii) induced a discontinuous and altered cuticle in some regions while epidermis was often thinner or missing; iii) rendered chitin cuticle presence discontinuous and less evident. In both control and novaluron larvae, chitin was present in the peritrophic matrix. This study showed quantitatively and qualitatively evidences of novaluron effects on *Ae. aegypti* larval development. To our knowledge, this is the first report describing histological alterations produced by a BPU in immature vector mosquitoes.

## Introduction

The mosquito *Aedes aegypti*, an important vector of arboviruses such as dengue fever, urban yellow fever and chikungunya [1], [2] is a holometabolous insect possessing a life cycle with four stages: egg, four larval instars, pupa and adult. Being fundamentally aquatic, this mosquito reaches the terrestrial environment only as an adult [3]. Mosquitoes in all stages of post-embryonic life, like any insect, have their bodies covered with an integument composed of an innermost epidermal monolayer and an outermost complex extracellular matrix called cuticle [4]. Proper growth and development after egg hatching requires periodic molting which begins with apolysis, followed by production of a new cuticle and ending with ecdysis, the shedding of the old cuticle *per se* [3], [4].

Insect molting depends, among other factors, of a precise interplay between biosynthesis and degradation of chitin, a polysaccharide composed of N-acetylglucosamine residues and regarded as one of the major components of the insect cuticle [5], [6]. In mosquitoes, chitin is present in the integumental cuticle and peritrophic matrix of larvae and adults and in the serosal cuticle of eggs [7], [8].

Concerning *Ae. aegypti* control, in addition to the recommended mechanical elimination of breeding sites, those permanent recipients that cannot be discarded are generally treated with chemical insecticides. These compounds, that largely target the insect's central nervous system, are applied against immature stages in water being also sprayed against adults, mainly during dengue outbreaks [9], [10]. The intensive use of neurotoxic insecticides for decades culminated in the loss of effectiveness due to resistance acquisition in several vector populations [11]–[13]. Therefore, novel substances with different target sites have been evaluated for *Ae. aegypti* control as well as for other arthropod vectors and agricultural pests. Among these substances, Insect Growth Regulators such as chitin synthesis inhibitors (CSI) have been tested with promising results [14]–[16]. The CSIs are, mostly, compounds belonging to the benzoyl-phenyl-urea (BPU) class which were discovered in the 1970s and affect chitin biosynthesis, cuticle formation and the molting process [14], [17]. BPUs act in larvae and pupae, hampering survival for the next molting [18], [19].

More than forty articles in the literature show BPUs efficacy for mosquito vector control (e.g: [18] and references therein, [20]–[24]), including populations that are resistant to neurotoxic insecticides [25]. However, only few investigations followed the outcome of the treatment and reported that adults surviving BPU treatment were physiologically debilitated [24], [26]–[30]. Additionally, even fewer studies describe the direct effects of BPU treatment on the immature stages of mosquitoes [26], [31]–[33].

In Brazil, the National Dengue Control Program, from the Ministry of Health (PNCD/MS) recommends, in case of resistance to neurotoxic insecticides, the use of alternative larvicides already evaluated by PNCD/MS (i.e. tested under Brazilian climatic and operational conditions) and approved by WHO for use in potable water. The BPU novaluron is, among others, one such compound [34], [35]. Due to the effectiveness of BPUs and their current use in *Ae. aegypti* control, it becomes increasingly necessary to characterize BPU-induced physiological alterations on this vector. This study has a dual role, first to understanding different aspects of the physiology of *Ae. aegypti's* larval molting process and second, to show novaluron effects on instar duration, chitin content and the structure of both the integument and internal tissues of developing mosquito larvae.

## Methods

### 1. Mosquito rearing and synchronous development of larvae

*Aedes aegypti* mosquitoes from the insecticide-susceptible Rockefeller strain were used in all tests. Adults were kept at 25±1°C and 70–80% r.h. [8]. To obtain synchronized developing larvae, eggs from colony stocks were used. For each experiment approximately 2,000 eggs were stimulated to hatch for 30 minutes in 200 mL plastic cups with 100 mL of rearing water, inside a B.O.D. incubator at 28±0.5°C. After 30 minutes, groups of 500 first instar larvae were transferred to plastic bowls containing 1 L of dechlorinated water and 1 g of cat food (Friskies®, Purina, Camaquã, RS, Brazil) and kept inside a B.O.D. incubator at 28±0.5°C until the third instar was reached. Larvae were then used in assays in a climatized room (see section 2) or inside a B.O.D. incubator (see sections 3–7).

### 2. Analysis of development and viability parameters

Tests described in this section were performed in a climatized room with a less precise temperature control (26±2.0°C) than a B.O.D. incubator. In parallel to the novaluron bioassays performed inside a B.O.D. incubator (described below in section 5), four cups with 10 larvae each were prepared under the same conditions of novaluron bioassays (control, EI_50_ and EI_99_) for each experiment. These simultaneous samples were followed with three aims; i) confirm emergence inhibition rates as indicated by probit analysis, ii) evaluate novaluron action over each instar or stage duration period and iii) evaluate novaluron action over emerging adults (males and females) percentage in the partially lethal EI_50_ concentration. All tests were monitored until all adults emerged or until no live larvae or pupae were observed. At least three experiments were performed.

Since these tests were performed under slightly different temperature conditions from the bioassays (section 5), it is not feasible to directly compare results shown in Section 2 of Results (linked to this section of Methods) with those presented in Section 3 of Results (related to bioassay conditions, as described in sections 3, 4 and 5 below).

### 3. Follow up of larval instar duration

The follow up of larval instar period duration was done by counting exuviae eliminated during the molting process [3]. Larvae reached the third instar inside plastic bowls at 48 hours after hatching (HAH) (see Results) when they were transferred to 300 mL transparent plastic cups at the density of 10 larvae/cup, containing 150 mL of dechlorinated water and approximately 0.15 g of cat food. Third instar larvae (L3) were periodically observed at hourly intervals until the first exuviae came out, indicating the emergence of the earliest fourth instar larvae (L4). At this moment larval development was monitored every two to four hours until the earliest pupae emerged. Exuviae arising of novaluron treated larvae (see section 5) was also followed from the third instar. Cups were kept in a B.O.D. incubator at 28±0.5°C.

### 4. Third and fourth instars subdivision: definition of collection time points

One of the aims of this work is related to the molting process of larval instars L3 and L4, which involves a sequence of biochemical events related to cuticle production and disposal. It was necessary to stage L3 and L4 in order to define experimental points representing the beginning, middle and end of each instar, to be used in subsequent analysis. Definition of sample collection time points was based on intervals between sequential molts and on control larva morphological characteristics observed before and after ecdysis [3]. The “early larvae” (L3e or L4e) were collected five hours after ecdysis from previous instar, “late larvae” (L3l or L4l) were collected two hours before ecdysis to the next instar (or stage) (see Results) and “intermediate larvae” (L3int or L4int) were collected in the middle of each larval period. Due to technical constrains, L4int larvae were collected with a 7.5 hours delay (see Results). The time points employed for novaluron-treated larvae collection were the same as those used for control larvae raised under physiological conditions.

### 5. Novaluron bioassays with synchronized larvae

Newly hatched 3^rd^ instar larvae (see section 1), were carefully moved from bowls to cups with the aid of a Falcon cell strainer (70 µm Nylon, BD Biosciences catalog # 352350). Inside the cups, larvae were continuously exposed to novaluron (Rimon® 10 EC, Figure S1) as previously described [25]. Cups were kept inside a B.O.D. incubator at 28±0.5°C with a controlled photoperiod (12 hours light/12 hours dark). Novaluron concentrations employed were defined by probit analysis matching emergence inhibition (EI) of 50 and 99% adults: 0.10–0.14 µg/L for EI_50_ and 0.30 µg/L for EI_99_. Controls were performed with 90 µL of acetone (novaluron solvent) added in each cup, equal to the acetone volume used for the novaluron EI_99_ concentration. In each bioassay and for each condition, 24 cups with 10 larvae and 150 mL per cup, prepared as described above, were used. Due to technical constrains, L3 larvae were exposed to the insecticide from 51 HAH (3 hours after L2 to L3 ecdysis, see Results) on. Larvae from control and experimental groups were collected in the previously defined time points (L3e, L3int, L3l, L4e, L4int and L4l, see section 4 above) when they were processed for chitin quantification or histological analysis (sections 6 and 7). All assays were repeated at least three times.

### 6. Chitin quantitation

Novaluron effect over chitin content was analyzed in 15 larvae exposed to each condition (control, EI_50_ and EI_99_), in each test. These larvae were collected at the time points defined previously, L3e to L4l, and stored without water at −70°C until use. Chitin content was evaluated by quantification of glucosamine derivatives obtained by deacetylation, depolymerization and deamination of the N-acetyl-glucosamine polymer as previously described [32], [36]. Briefly, chitin undergoes an alkaline digestion and is converted by deacetylation to chitosan (i.e., glucosamine polymer), through the joint action of high temperature (130°C) and high alkaline concentration (14 M KOH). Aldehydes derived from depolimerized chitosan and deaminated glucosamine, generated in a reaction with HNO_2_ and with the further addition of MBTH and Fe^+3^, were measured colorimetrically at 650 nm. Chitin content was expressed as glucosamine equivalents, according to a standard curve obtained with commercial glucosamine (Sigma-Aldrich, catalog # G -4875). Before chitin quantification, larvae weight was determined to normalize the results.

### 7. Histological analysis

Since the effect of other BPUs is directly related to larval exposure time to the product [25], greater effects were expected to be found on L4l larvae after novaluron treatment. Control and EI_99_ L4l larvae were fixed in Fornoy (60% ethanol, 30% formaldehyde 37% and 10% glacial acetic acid) and stored at 4°C for up to five days. In order to avoid interference of after-death morphological alterations only alive larvae were collected for morphological analysis.

#### 7.1. Histochemical processing

Fixed larvae were subjected to serial washes of 10 minutes each in solutions of 70, 80 and 90% ethanol followed by two washes of 10 minutes each in 100% ethanol and immersion in 100% ethanol for 40 minutes. Samples were passed through three washes in xylene (10, 10 and 40 minutes) and were embedded in Paraplast Plus® (Sigma-Aldrich, catalog # 76258) at 60°C for 24 hours. Serial microtome 7 µm thick sections were obtained for the mesothorax region, being collected on slides which were subsequently stained with hematoxylin and eosin (HE) or labeled with the lectin WGA coupled to FITC (see below).

The mesothorax region was chosen due to the presence of gastric cecae [3] and previous results showing abnormalities caused by BPU in this region [26]. The interpretation of internal larval structures at the mesothorax region was determined through a careful and detailed reading of chapters 9, 10, 13 and 14 of the *Aedes aegypti* book by Christophers [3].

#### 7.2. Hematoxylin and eosin (HE) staining

In order to remove paraffin, slides containing larvae sections were bathed in the following series of solutions: xylene (2 washes of 5 minutes each), 100% ethanol (two washes of 3 min each), 95% ethanol (3 min), 70% ethanol (3 min) and distilled water (3 min). The HE staining was initiated with a bath in hematoxylin solution (Fisher Scientific, catalog # BP2523) (45 seconds), followed by tap water, Scott water (10 g of MgSO_4_, 2 g of NaHCO_3_ in 1 L distilled water) (2 min), distilled water (2 min), 70% ethanol (3 min), eosin solution (Polysciences, Inc, catalog # 09859) (2 min), 95% ethanol (30 seconds), 100% ethanol (2 washes of 30 seconds each) and xylene (2 washes of 30 seconds each) [37]. After xylene bath, slides were mounted with cover slips, using Entellan® (Merck, catalog # 100869). Sections were examined under an Axioplan (Zeiss) microscope with bright field or differential interference contrast (DIC) and images were captured with the digital camera AxioCam HRC (Zeiss).

#### 7.3. Chitin labeling

Paraffin was removed from slides exactly as described above. Slides were then washed 3 times in PBS buffer containing 2 mg/mL BSA (PBSB). Slides were then incubated in PBSB solution with 100 µg/mL WGA-FITC (EY Laboratories) for 40 minutes. Slides were then washed three times with PBSB and mounted with the anti-fade Fluor Save™ Reagent (Calbiochem, catalog # 345789). Sections were examined under a fluorescent microscope Axioplan (Zeiss), FITC channel, with ratio of absorption/excitation 492 nm and emission at 517 nm. WGA (wheat germ agglutinin) is a lectin highly specific for N-acetylglucosamine polymers [38], [39].

### 8. Statistical analysis

For all experiments, mean and standard deviation were calculated. One way analysis of variance (ANOVA) (P<0.05) followed by Bonferroni multiple comparison test was used in chitin quantification analysis. In the analysis of emergence inhibition and percentage of adults (males and females) after novaluron treatment, a one way ANOVA followed by Kruskal-Wallis test (P<0.05) was performed.

## Results

### 1. Novaluron inhibits adult emergence and alters adult sex ratio

**Figure 1:**
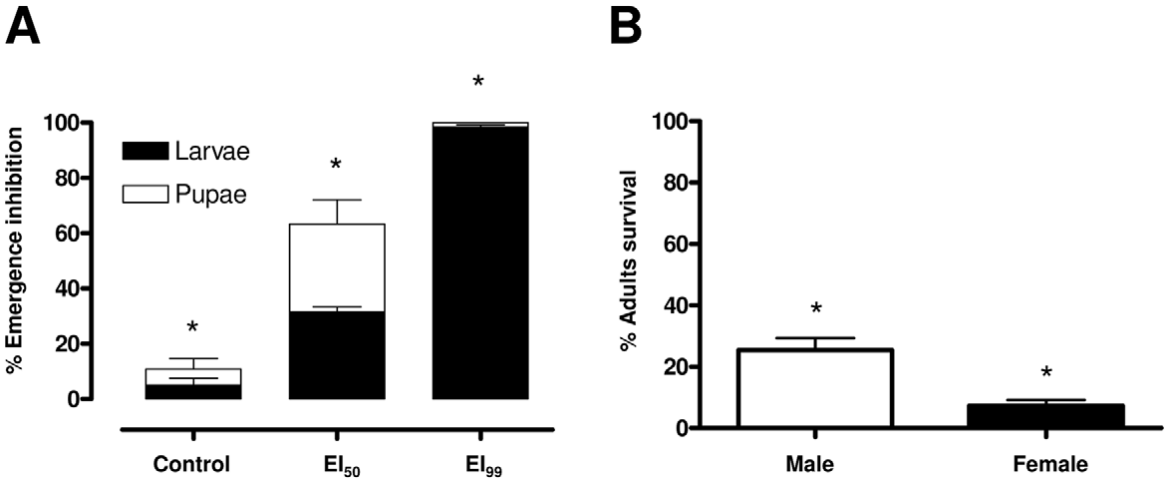
Novaluron inhibits *Ae. aegypti* adult emergence. (**A**) Dose-dependent effect of novaluron over emergence inhibition. EI_50_ and EI_90_ indicate novaluron concentrations resulting in emergence inhibition of 50 and 99% of adults, respectively. Black and white bars indicate death at larval and pupal stages, respectively (**B**) Percentage of surviving adults (males and females) after novaluron treatment (EI_50_). Bars indicate mean and standard deviation of three experiments. Asterisks indicate significant differences (ANOVA, P<0.05).

Novaluron (Figure S1) inhibited adult emergence and induced mortality in a dose-dependent manner (Figure 1). Besides this effect, a direct relationship between novaluron concentration and earlier mortality of immature specimens was observed: the proportion of dead larvae increased on EI_99_ compared with EI_50_ (Figure 1A). Adults that survived novaluron treatment (EI_50_) emerged mostly as males (ANOVA; P<0,05) (Figure 1B).

### 2. Novaluron delays development of immature specimens

**Figure 2:**
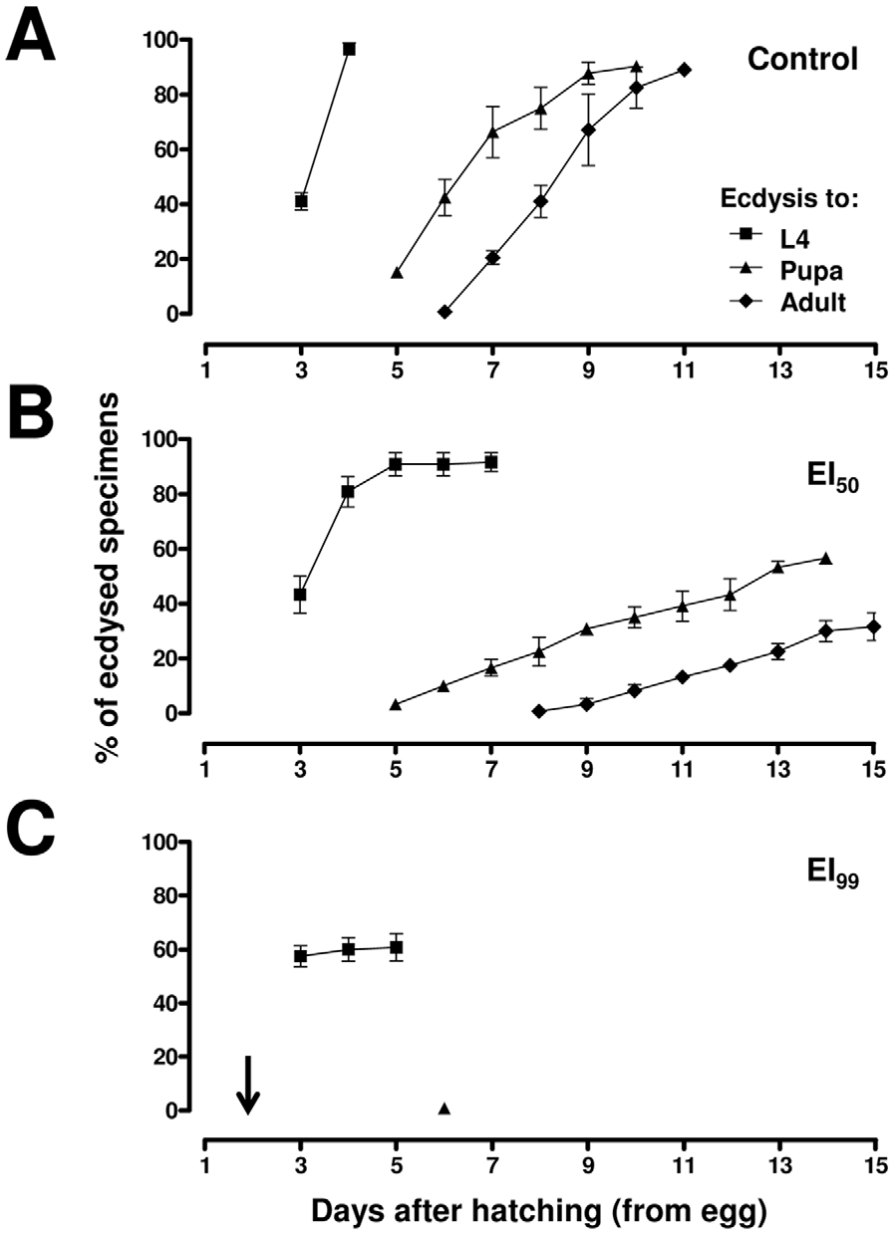
Novaluron induces delay in the development of *Ae. aegypti* immatures. Symbols represent the cumulative percentage of specimens in relation to eliminated exuviae of the preceding instar: squares, triangles and lozenges indicate newly emerged L4, pupae and adults, respectively. (**A**) control; (**B**) EI_50_ and (**C**) EI_99_. Bars represent the standard deviation of three independent experiments. Arrow indicates the moment of novaluron administration (see Methods).

The experiments described in this section were performed to confirm whether novaluron affects the duration of immature stages, before adult emergence, as evaluated in bioassays (Figure 2). As stated in Methods, these experiments were conducted in a climatized room, with a slightly less precise temperature adjustment than B.O.D. incubators, used in further experiments. Under control conditions (Figure 2A), about 40% of larvae were at L4 on the 3^rd^ day after hatching and all specimens became L4 on the 4^th^ day. Ecdysis to pupa took place between days 5 and 10 and adult emergence occurred between days 6 and 11 after hatching. When the novaluron EI_50_ was used (Figure 2B), larvae ecdysis to L4 was also initiated on the 3^rd^ day but only on the 7^th^ day it was completed. Pupa ecdysis also began on the 5^th^ day but was prolonged until the 14^th^ day and adults emerged between days 8 and 15. With the highest novaluron condition, EI_99_ (Figure 2C), L4 larvae ecdysis began on the 3^rd^ day and finished between days 4 and 5. However, only 60% of the larvae succeeded in completing the entire molting process, reaching L4. Only 2.5% of the specimens reached the pupal stage and no immature became adult.

### 3. Larval instars duration at 28°C

**Figure 3:**
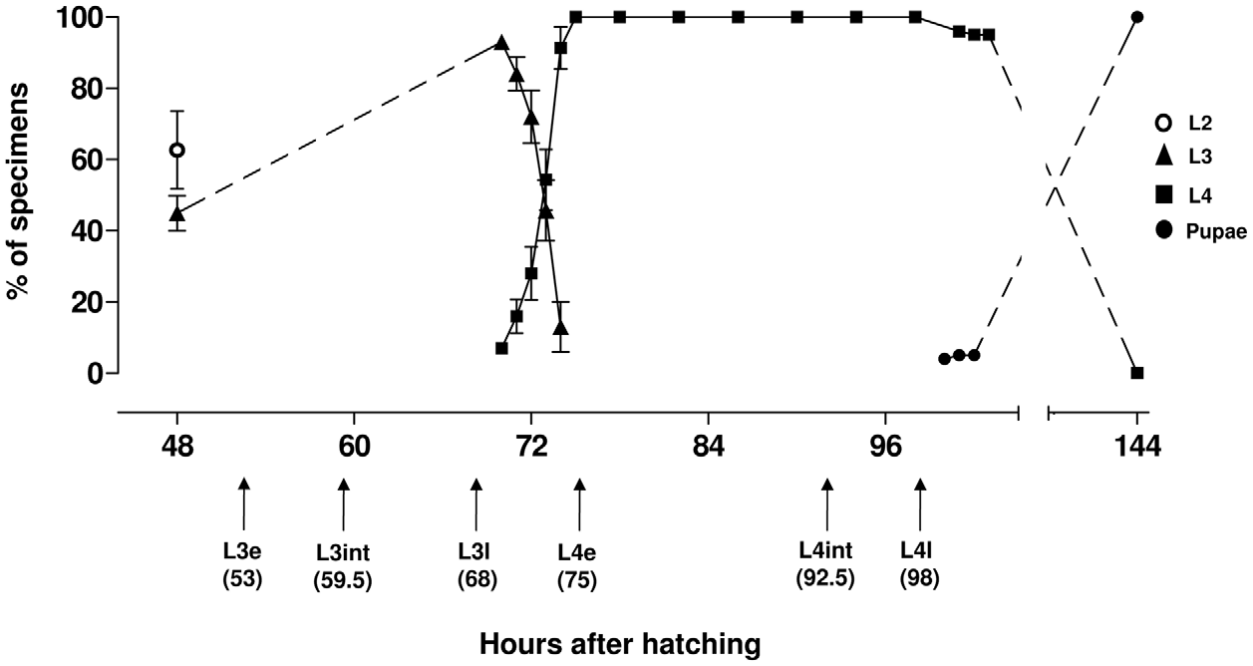
Period duration of *Ae. aegypti* larval ecdysis. Symbols represent the cumulative percentage of specimens at different immature stages. Bars represent the standard deviation of three independent experiments. Arrows indicate the experimental points, defined in hours, as early (e), intermediate (int) and late (l) moments for each instar.

In order to perform chitin quantitation and histological analyses in developing L3 and L4 larvae, it was necessary to subdivide these stages to define experimental points representing the beginning, middle and end of these instars. Therefore, a follow up of larval instar period duration was performed from the end of L2 until pupation (Figure 3). The beginning of L2 to L3 ecdysis occurred 48 hours after hatching (HAH) and, due to synchronic L2 larvae development, about 45% of the specimens reached the third instar simultaneously. From this point on, a follow-up of larval instar period duration was performed from L3 until pupation of all individuals. In our conditions, a synchronous ecdysis occurred at L3 to L4 where the first L4 emerged at 70 HAH while five hours later (75 HAH) all specimens were at the fourth instar. On the other hand, L4 to pupa ecdysis was asynchronous: while the first pupae appeared at 100 HAH, the last specimens pupated 44 hours later (144 HAH). All these ecdysis periods (L2 to L3 at 48 HAH, L3 to L4 at 70 HAH and L4 to pupa between 100 and 144 HAH) are in accordance with descriptions made by Christophers [3]. The third instar lasted 22 hours (between 48 and 70 HAH) and the minimum and maximum period duration of the fourth instar was 30 hours (between 70 and 100 HAH) and 74 hours (between 70 and 144 HAH), respectively. In order to perform biochemical analysis of control, EI_50_ and EI_99_ larvae, early (e), intermediate (int) and late (l) L3 and L4 experimental points were collected according to developmental timing of control larva (i.e. L3e, L3int, L3l, L4e, L4int e L4l being respectively 53, 59.5, 68, 75, 92.5 and 98 HAH). Histological analyses were performed on alive L4l from both control and novaluron-treated conditions. See Figure 3 and Materials and Methods, section 4 for details.

### 4. Novaluron exposed larvae have low chitin content

**Figure 4:**
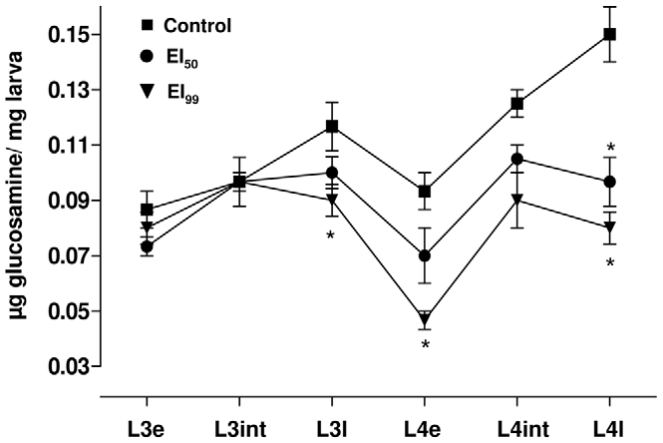
Effect of different novaluron concentrations on chitin production profile over *Ae. aegypti* L3 and L4 instars. Experimental time points are indicated horizontally. Symbols refer to the average value and standard deviation of three independent experiments. Asterisks indicate significant differences when compared to the control condition at the same experimental time point (ANOVA, P<0.05). Each sample was collected on time points defined for the physiological instar development of the control group, according to Figure 3 (see section 4 of Methods).

Chitin content was evaluated during L3 and L4 instars in control and novaluron exposed samples (Figure 4). There was an increase in chitin content during both the 3^rd^ and 4^th^ instars of control larvae. Although not significant (P>0.05) early 4^th^ instar (L4e time point) presented a chitin content below that of late 3^rd^ instar (L3l). It is important to highlight that the production of a new cuticle precedes the elimination of the old one (ecdysis) and the results found here seem to reflect the fact that L3l is a pharate larvae possessing two chitinous exoskeletons [3], [4]. Larvae exposed to novaluron EI_50_ and EI_99_ showed a general chitin content profile similar to the controls, with exception of late instars. Two distinct analysis of variance were performed. The first (not represented in Figure 4) compared values of every time point with the first one obtained (L3e) at each corresponding experimental condition. In control larvae, intermediate and late L4 chitin content was significantly higher than L3e (P<0.05). In novaluron EI_50_ and EI_99_ conditions, chitin content in all time points were equivalent, with exception of the EI_99_ early 4^th^ instar (L4e) content, being significantly lower than EI_99_ early 3^rd^ instar (P<0.05). The second analysis of variance was performed to verify novaluron's dose-dependent effect on chitin formation comparing, at each experimental point, the control, EI_50_ and EI_99_ situations (asterisks in Figure 4). Chitin production in the third instar was affected only in late EI_99_ larvae that exhibited diminished chitin content. During the fourth instar, early EI_99_ and late larvae of both novaluron conditions showed lower amounts of chitin when compared to control samples.

### 5. Novaluron affects larval tissues and the integument

**Figure 5:**
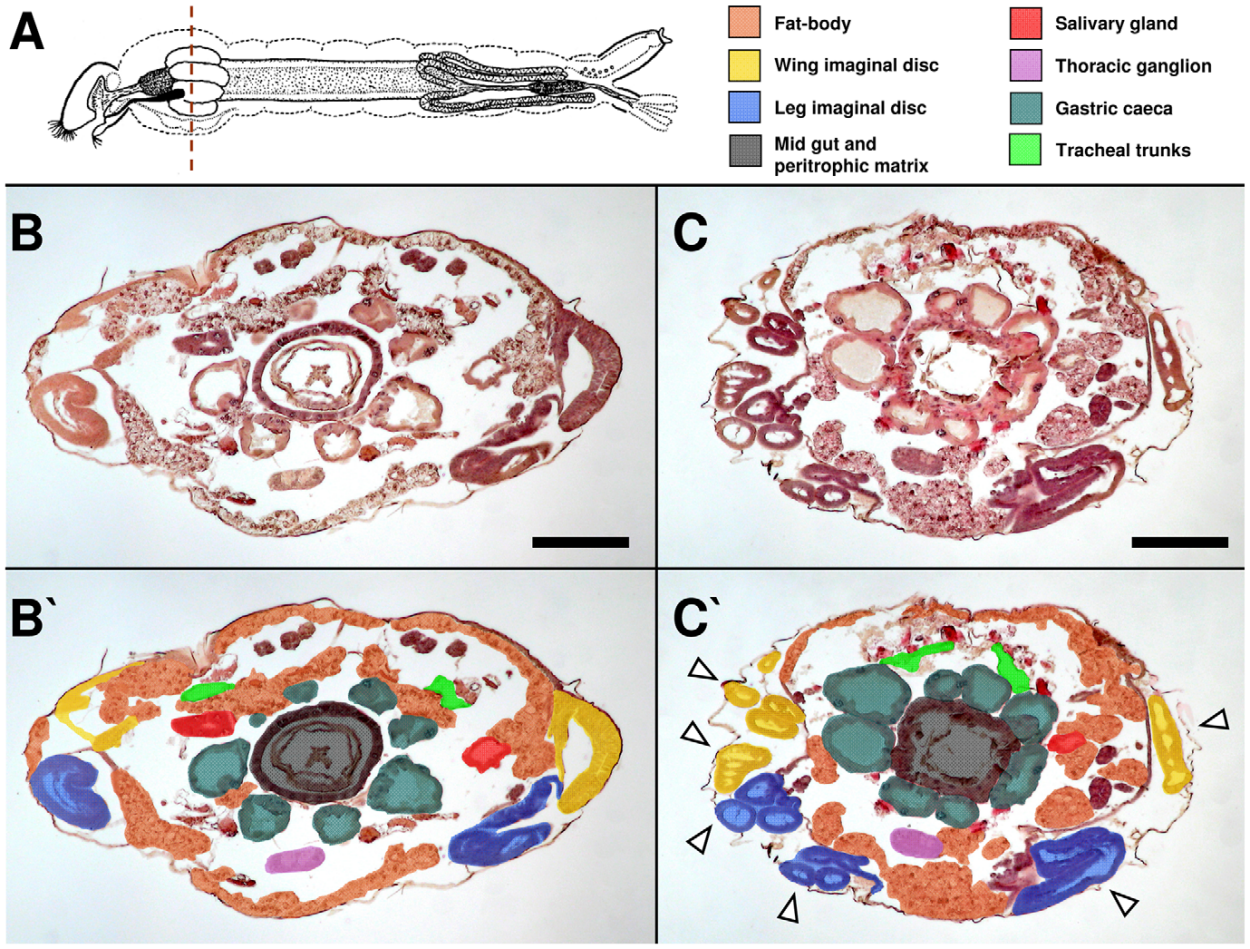
Novaluron induces histological alterations on *Ae. aegypti* larvae. Bright field microscopy. (**A**) Larva scheme with histological section region evaluated (mesothorax, dashed line) and color caption for identified organs and tissues. HE staining of live L4l larvae from control (**B**, **B′**) and novaluron EI_99_ (**C**, **C′**) are shown. In (**B′**) and (**C′**) sections shown in the corresponding panels were colored to better identify structures. In (**C′**) arrowheads indicate disorganized imaginal discs (see Figure S2 for further details). In (**A**), larva scheme adapted from Christophers [3]. Bar = 200 µm.

Serial cross sections of the mesothorax region were analyzed in alive late L4 larvae (L4l) in order to check for novaluron effects on internal organs and larval integument (Figure 5 and Figure S2). The mesothorax region was chosen since it is easily identified by the presence of gastric cecae (Figure 5A), also harboring several other organs and tissues that could be potentially affected by BPU treatment [26]. In control larvae, HE staining shows several mesothorax structures (Fig. 5B and B′). Immediately below the epidermis lies the fat body parietal layer located in the most dorsal and ventral regions, while the fat body visceral layer lies as lobes above salivary glands and the four most dorsal gastric caeca. Dorsal tracheal trunks are located above salivary glands and close to the most dorsal gastric caeca. Right above the middle line of the dorso-ventral axis are wing imaginal discs and right below the middle line, in the ventral region, are the other four gastric caeca, leg imaginal discs and the ventral nervous system (thoracic ganglion). Internally to the gastric caeca lies the midgut and within it, the peritrophic matrix. In novaluron treated larvae (Figure 5C and C′), fat body, salivary gland, tracheal trunks, gastric caeca, thoracic ganglion, midgut and peritrophic matrix are also observed, although their morphology are altered. Wing and leg imaginal discs are highly disorganized, not bearing a continuous tissue. See also Figure S2 for further details.

**Figure 6:**
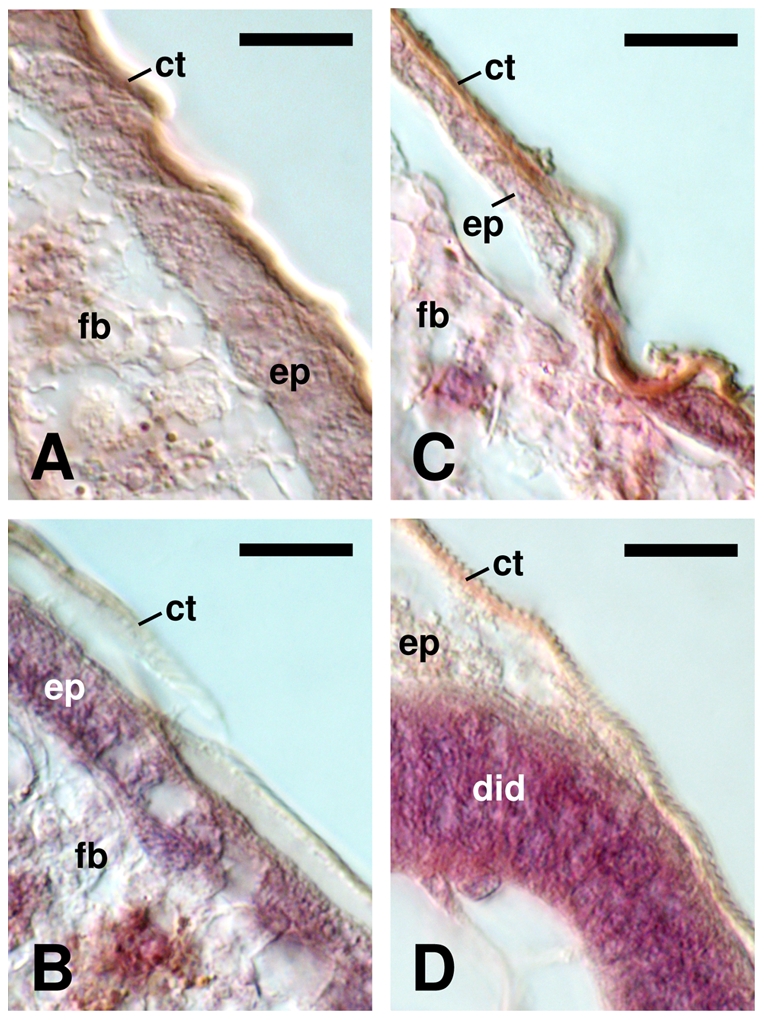
Novaluron modifies cuticle and epidermis aspect of *Ae. aegypti* larvae. DIC microscopy was performed on histological sections of late L4 larvae stained with HE. (**A**) Control. Note the close association among cuticle, epidermis and the subjacent fat body layer. (**B–D**) Novaluron EI_99_. Cuticle presents a semitransparent and discontinuous aspect being detached from the epidermis (**B**); epidermis is thinner (**C**) or degenerated, with a rope-like cuticle (**D**). ct: cuticle, did: disorganized imaginal disc, ep: epidermis, fb: fat body.

Regarding the integument of L4l larvae (Figure 6), in control samples the cuticle is closely associated with epidermis and the underlying fat body parietal layer can be observed (Figure 6A). In contrast, novaluron treated L4l larvae, show in some regions a translucent and discontinuous cuticle that is detached from the epidermis (Figure 6B) or a thinner epidermis (Figure 6C); in some cases the epidermis seems to be degenerated and the cuticle is altered, presenting a rope-like structure (Figure 6D).

### 6. Novaluron affects the presence of cuticular chitin

**Figure 7:**
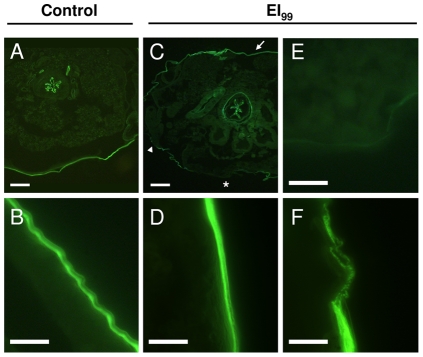
Novaluron alters cuticular chitin presence in late L4 larvae. WGA-FITC labeling was used to detect chitin by fluorescence microscopy. (**A**, **B**) Control larvae exhibiting continuous cuticle labeling. (**C–F**) Novaluron EI_99_ larvae show uneven cuticle labeling. Chitin labeling of cuticle in distinct regions of the larva is rather uniform (arrow in **C**; panel **D**), absent (asterisk in **C**; panel **E**) or irregular (arrowhead in **C**; panel **F**). Peritrophic matrix chitin labeling was not altered (**A**, **C**). Bar = 100 µm in **A**, **C** and 10 µm in **B**, **D–F**. All images were also recorded in bright field, in order to assure that images were in focus and that a cuticle was present (data not shown).

Labeling with wheat germ agglutinin (WGA), a lectin that is highly specific for N-acetyl-D-glucosamine polymers [38], [39], was performed to evaluate novaluron's effect in the presence of chitin in late L4 larvae (L4l) (Figure 7). WGA labeling was continuous throughout control larvae cuticle (Figure 7A, B) and was also observed in the peritrophic matrix (Figure 7A). Novaluron treated L4l cuticle labeling was uneven (Figure 7C–F), where some cuticle regions showed uniform labeling (Figure 7D), irregular labeling (Figure 7F) or no WGA labeling (Figure 7E). In these larvae, peritrophic matrix chitin labeling was not affected by novaluron treatment (Figure 7C).

## Discussion

Despite recent advances in studies regarding the cuticle and the physiological and biochemical processes related to its production and/or shedding [6], literature about this topic is still scarce when compared to current descriptions of other aspects of insect biochemistry and physiology [40]. Insect larvae exposed to chitin synthesis inhibitors such as the benzoylphenylureas (BPUs) develop fragile cuticles unable to support the increased tension during the molting process. Such larvae have difficulty to shed their exuviae, dying due to starvation, suffocation or even rupture of the weak, malformed cuticle [15], [17], [26]. Therefore, the study of BPUs effects on immature insect stages can also contribute to better understand the physiological process of cuticle formation and elimination. Compounds such as the BPU diflubenzuron and other Insect Growth Regulators have already been recommended by WHO Pesticide Evaluation Scheme (WHOPES) for use against aquatic mosquito stages, some of them even in drinking water [9], [34]. In Brazil the recent employment of BPUs in the national program of dengue control corroborates the need of a detailed knowledge about the potential effects and mechanisms of action of these compounds [21], [23].

Novaluron efficacy was confirmed in this work, as previously described [19], [35]. When exposed to a partial lethal concentration, surviving male and female emergence percentage differed significantly, occurring a higher percentage of male emergence (Figure 1), as already described for the BPU triflumuron [24]. This is attributed to the faster development of male, and their consequent shorter contact with the BPU (see below). The higher male emergence among novaluron exposed specimens is a parameter of epidemiological relevance since dengue transmission takes place through females.

Comparing to other BPUs, novaluron is very potent, inhibiting 99% of *Ae. aegypti* adult emergence in a concentration 6 to 12 times lower than other BPUs like triflumuron and diflubenzuron [25], [28]. We believe that, as diflubenzuron [35], [41], novaluron is a promising alternative to contribute for the control of the dengue vector and other epidemic diseases transmitted by mosquito in urban areas [34].

Novaluron EI_50_ prolonged the duration of *Ae. aegypti* immature stages, which was also described for the mosquito *Culex pipiens pipiens* with another BPU [31]. In contrast to EI_50_, in control larvae there was no overlapping in the timing of L3 to L4 ecdysis and L4 to pupa ecdysis. Larvae exposed to the novaluron EI_99_ dose died mainly before reaching L4 to pupa ecdysis. Curiously, at the 3^rd^ day after hatching, while 40% of control larvae had undergone L3 to L4 ecdysis, this rate was already 60% for EI_99_ larvae. We believe this difference might be due to the stress induced by the presence of novaluron. Evidences for such physiological stress were observed in the histological analysis (see below).

In insects the progression through larval instars or stages is easily defined by the molt, an event that finishes punctually when the exuviae from the previous phase is released [4]. However, larval growth within any given instar is continuous, with steady gain of weight and constant increase in size of non sclerotized cuticle regions [4]. *Aedes aegypti* larvae follow this pattern throughout development; e.g. a L3 larvae exhibits a weight ratio increase of 4.7 and a volume ratio increase of 4.3 when early (right after L2 to L3 ecdysis) and late (right before L3 to L4) L3 specimens are compared [3]. Due to this continuous growth of developing larvae, which is a dynamic and complex process, a larva at the beginning of an instar is a physiological entity distinct from a larva at the end of the same instar. For this reason, it was necessary to follow the duration period of immature *Ae. aegypti* stages from L2 to L3 transition until pupation. This allowed the subsequent division of L3 and L4 in “early”, “intermediate” and “late” time points, to be used for the following analysis. Both L2 to L3 and L3 to L4 ecdysis occurred more synchronously that L4 to pupa ecdysis, corroborating the data described by Christophers [3]. The lower synchrony in L4 to pupa ecdysis can be attributed to the difference of developmental time requirements of each sex, that can be up to one day, with males emerging first [3], [7], [42].

In control larvae the chitin content increased gradual and significantly during each of the evaluated instars, L3 and L4. A reduction of 20% in chitin content was observed in early L4 when compared to late L3. This difference was already expected since at the end of each instar the insect larva has two cuticles, and the old one is lost at the end of the molting process [4]. Accordingly, Candy and Kilby [43] observed an increase in the chitin amount during the fifth instar of the grasshopper *Schistocerca gregaria* and a reduction at the beginning of the next stage.

In novaluron treated *Ae. aegypti* larvae, the chitin content was effectively reduced in a dose-dependent manner. Similar results were described for *Anopheles quadrimaculatus* third instar larvae exposed to diflubenzuron [32], [33].

Histological descriptions of *Ae. aegypti* larvae are scarce [3]. Moreover, to our knowledge this is the first report of histological analysis of internal larval alterations produced by any BPU in immature *Ae. aegypti*. The mesothorax region was chosen due to the presence of the easily recognizable gastric ceca [3] and since *Ae. aegypti* L4 larvae, when exposed to a BPU, show dorsal splitting and bulbous projection at the thorax [26], pointing out a sensibility in this region for chitin synthesis inhibitor treatments. We thus asked how novaluron would affect the internal structures of L4 larvae.

The methodology employed for histochemical fixation and processing preserved the exoskeleton and internal structures in both conditions tested (control and EI_99_). Novaluron administration interfered with the cuticle structure, showing a rope-like aspect in some regions. It also altered the chitin presence in some cuticle regions. Chitin labeling in the peritrophic matrix of the midgut remained unchanged with novaluron, confirming reports on chitin quantitation in *An. quadrimaculatus* larvae exposed to diflubenzuron [33]. Novaluron effects were similar to the phenotype of *Drosophila melanogaster* mutants for genes implicated in cuticle formation [6], [44], [45] and to the beetle *Tribolium castaneum* silenced for the *TcCHS1* gene, coding for the chitin synthase responsible for cuticular chitin synthesis [46].

Novaluron affected the epidermis and other internal larval organs. These systemic alterations demonstrate how debilitated larvae are after novaluron treatment. Although histological analyses were performed on alive animals, all EI_99_ larvae analyzed would probably die before completing ecdysis to pupae, since only 2.5% of specimens reach the pupal stage after EI_99_ treatment (Figure 2C). Novaluron seems to particularly disrupt the development of imaginal discs, since leg and wing imaginal discs are not properly formed in novaluron treated larvae. Accordingly, adults surviving BPU treatment with curved tarsi and crippled wings were already shown [24], [26].

Novaluron is among the few compounds presently recommended by WHO for use in drinking water against the main dengue vector, *Ae. aegypti* [34]. We confirmed the dose-response novaluron effect on adult emergence. Female mosquitoes, the epidemiologically relevant sex, are more affected than males. Additionally, we established a detailed protocol of synchronized rearing and quantified the chitin content along the development of strictly staged larvae. We demonstrated the interference of novaluron on cuticle deposition of chitin and confirmed the profile of chitin amount during the molting process. Finally, histological alterations in a series of mosquito organs and internal structures were identified on larvae exposed to this BPU. Besides potential of BPUs towards vector control, their use as a tool to unravel both the complex physiologic molting process and details of the exoskeleton structure and function should be envisaged, in order to reveal novel potential targets against mosquito vectors.

## Supporting Information

Figure S1 **Novaluron chemical information.** (A) Structure, (B) Molecular formula and (C) IUPAC name. Adapted from PubChem website (http://pubchem.ncbi.nlm.nih.gov/).
(PDF)

Figure S2 **Histological cross sections of control and novaluron-treated** ***Ae. aegypti*** **larvae at the mesothorax region.** The following slides present 45 histological cross sections of 4 control and 3 novaluron-treated (EI_99_) *Ae. aegypti* larvae at the mesothorax region. In all sections ventral side is down (i.e. the ventral thoracic ganglion is always at the bottom). Numbers in the panels denote the sequence of cross sections. For further details, please check Methods, section 7 and Figure 5 of the main text.
(PDF)
