# Supplementary material for: Physiological and Morphological Aspects of Aedes aegypti Developing Larvae: Effects of the Chitin Synthesis Inhibitor Novaluron
Source: PLoS One. 2012 Jan 24;7(1):e30363. doi: 10.1371/journal.pone.0030363 (PMC3265478; doi:10.1371/journal.pone.0030363)
Supplement: Figure S1 — Novaluron chemical information. (A) Structure, (B) Molecular formula and (C) IUPAC name. Adapted from PubChem website (http://pubchem.ncbi.nlm.nih.gov/). (PDF) [file pone.0030363.s001.pdf]

A

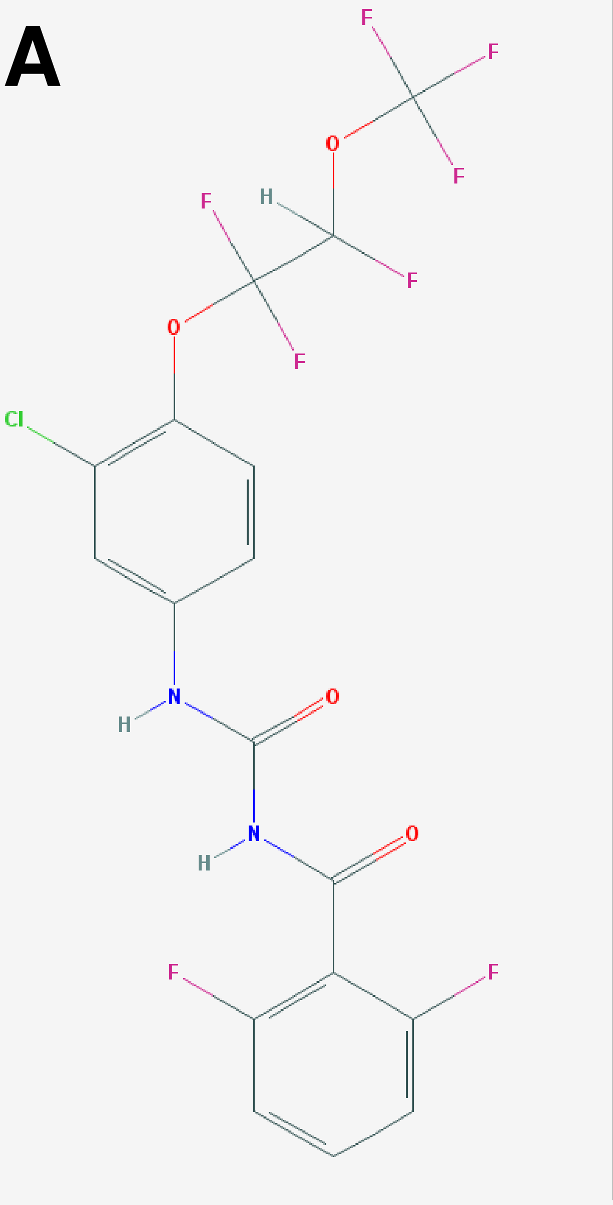

B

$C_{17}H_9ClF_8N_2O_4$

C

N-[[3-chloro-4-[1,1,2-trifluoro-2-(trifluoromethoxy)ethoxy]phenyl]carbamoyl]-2,6-difluorobenzamide

Supplementary Figure 1: Novaluron chemical information. (**A**) Structure, (**B**) Molecular formula and (**C**) IUPAC name. Adapted from PubChem website (http://pubchem.ncbi.nlm.nih.gov/).
