# Supplementary material for: Physiological and Morphological Aspects of Aedes aegypti Developing Larvae: Effects of the Chitin Synthesis Inhibitor Novaluron
Source: PLoS One. 2012 Jan 24;7(1):e30363. doi: 10.1371/journal.pone.0030363 (PMC3265478; doi:10.1371/journal.pone.0030363)

Supplementary Figure 2 – Farnesi et al (Physiological and morphological aspects of *Aedes aegypti* developing larvae: effects of the chitin synthesis inhibitor novaluron)

The following slides present histological cross sections of control and novaluron-treated (EI_{99}) *Ae. aegypti* larvae at the mesothorax region.

In all sections ventral side is down (i.e. the ventral thoracic ganglion is always at the bottom).

For further details, please check Methods, section 7 and Figure 5 of the main text.

Number in the panels denote the sequence of cross sections.

The next 6 slides show cross sections of 4 control larvae at the L4l stage.

First control L4l

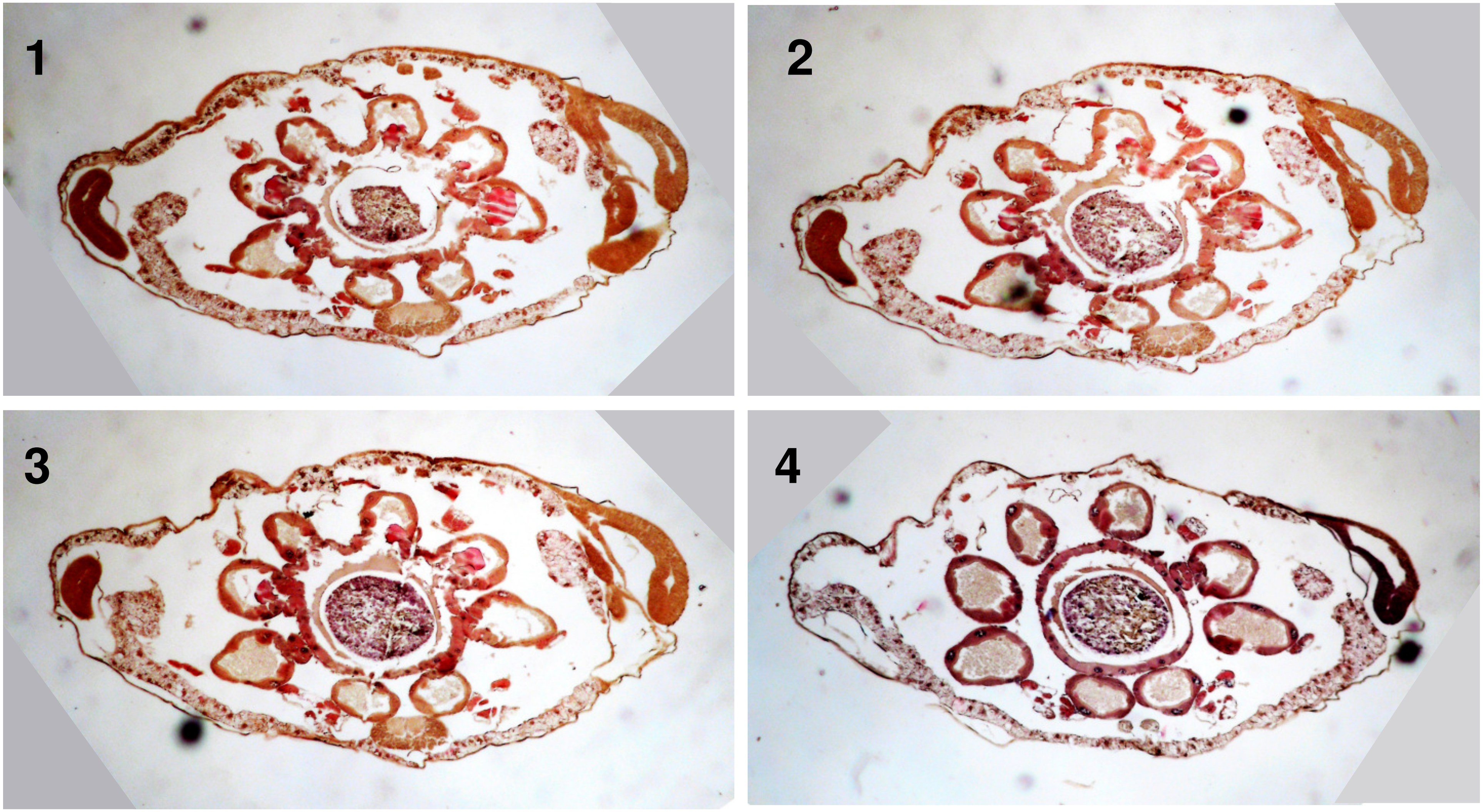

First control L4I (continued)

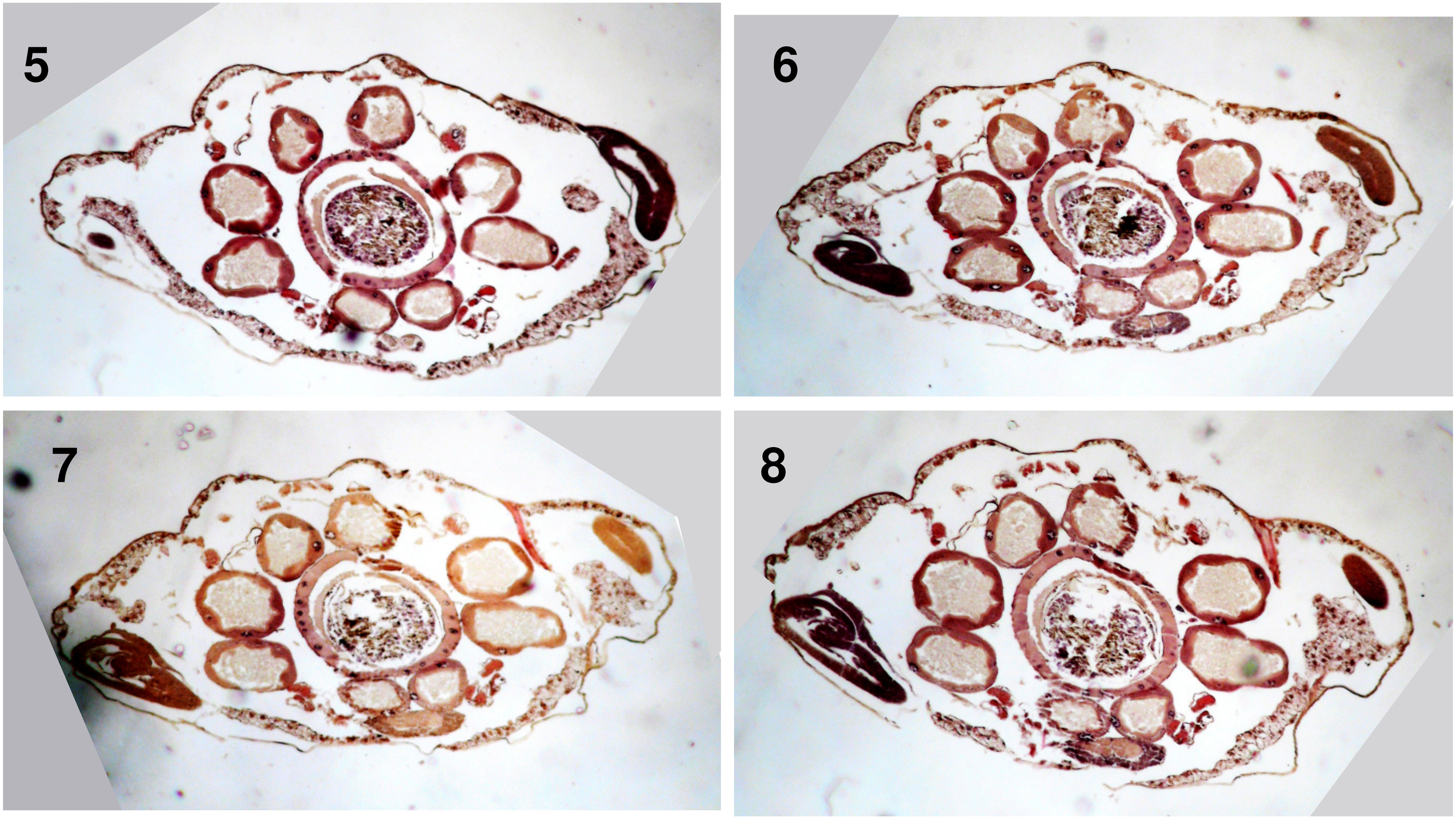

First control L4I (continued)

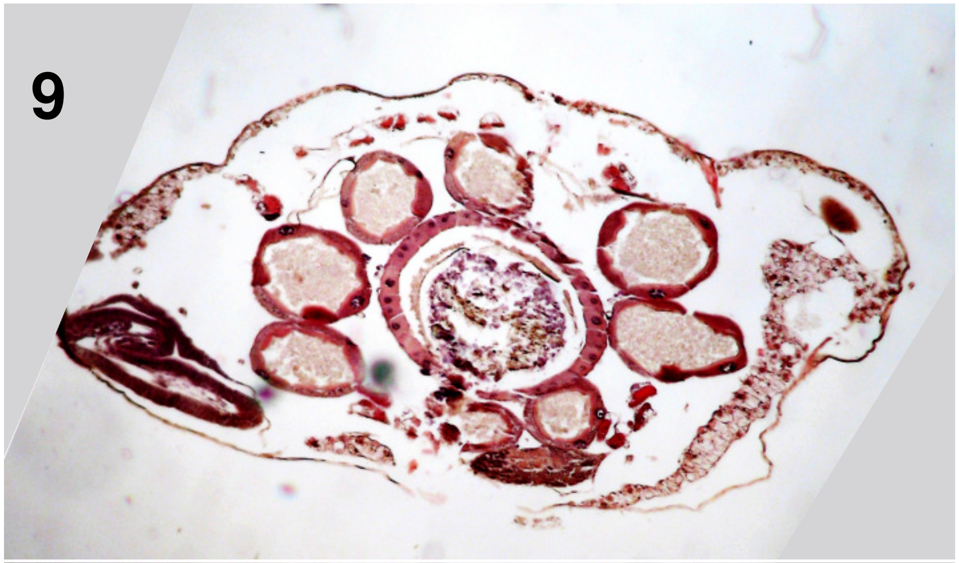

Second control L4I

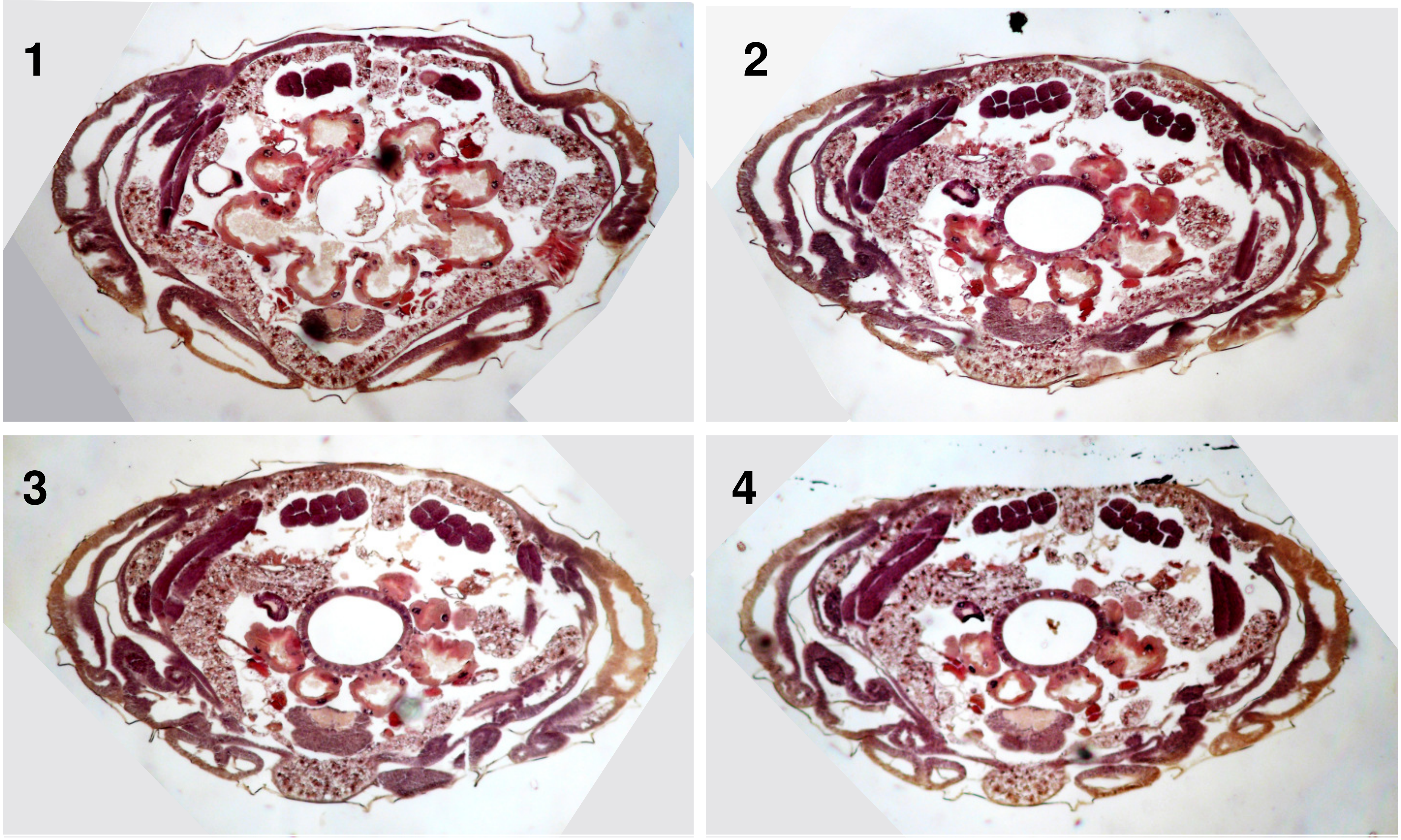

Third control L4I

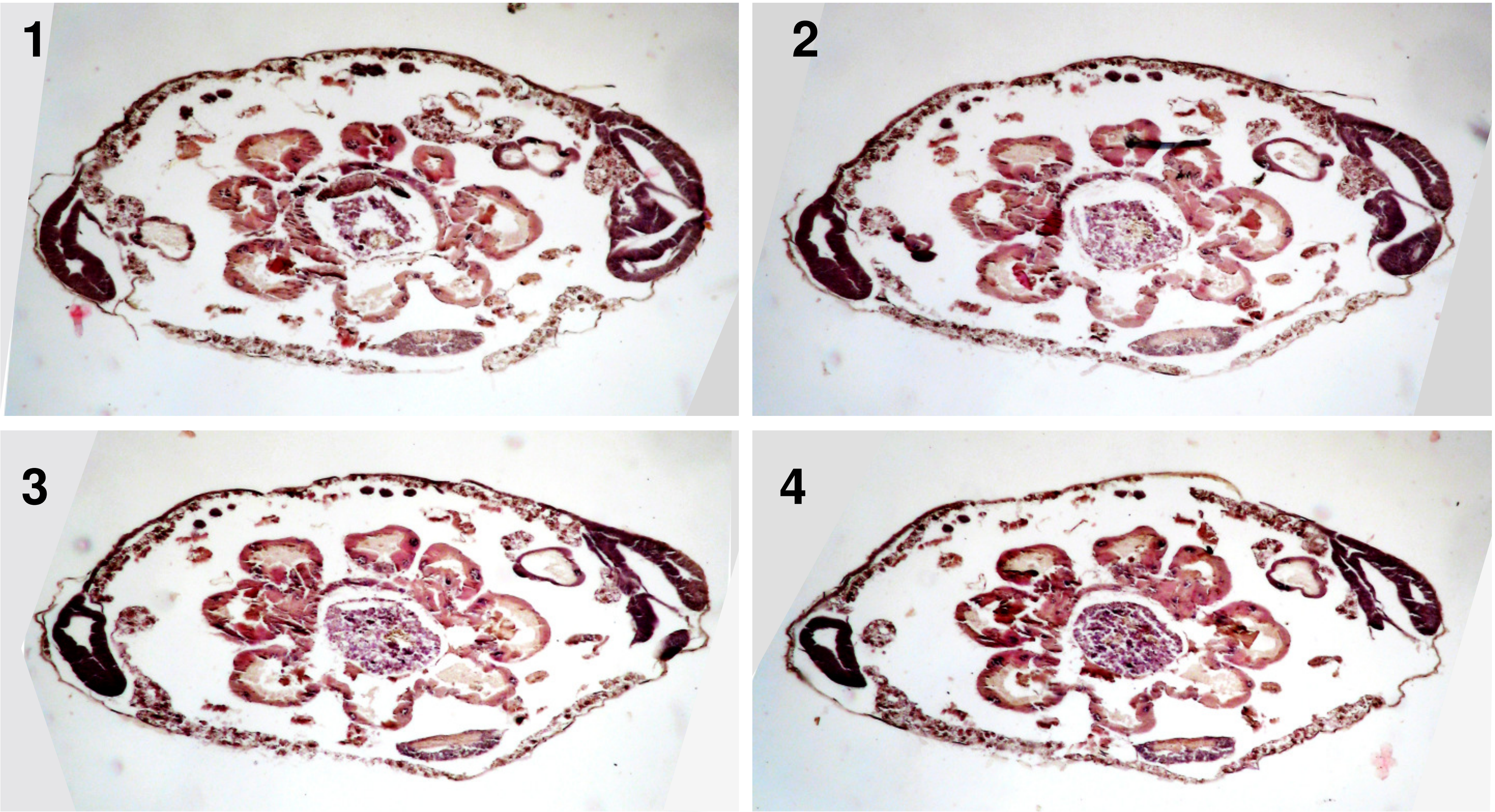

Fourth control L4I

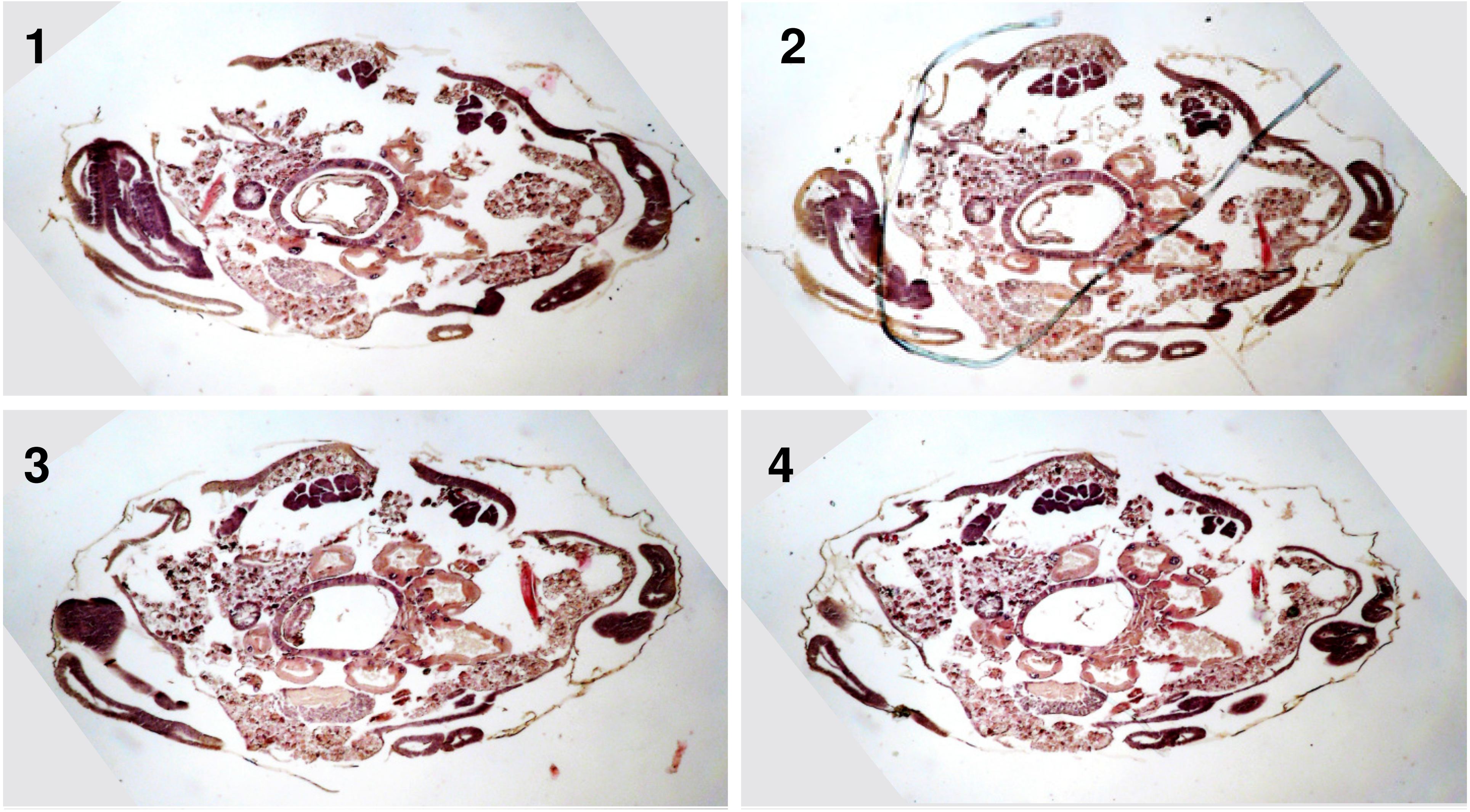

The next 6 slides show cross sections of 3 novaluron-treated larvae (EI_{99}) at the L4l stage.

First Novaluron L4I

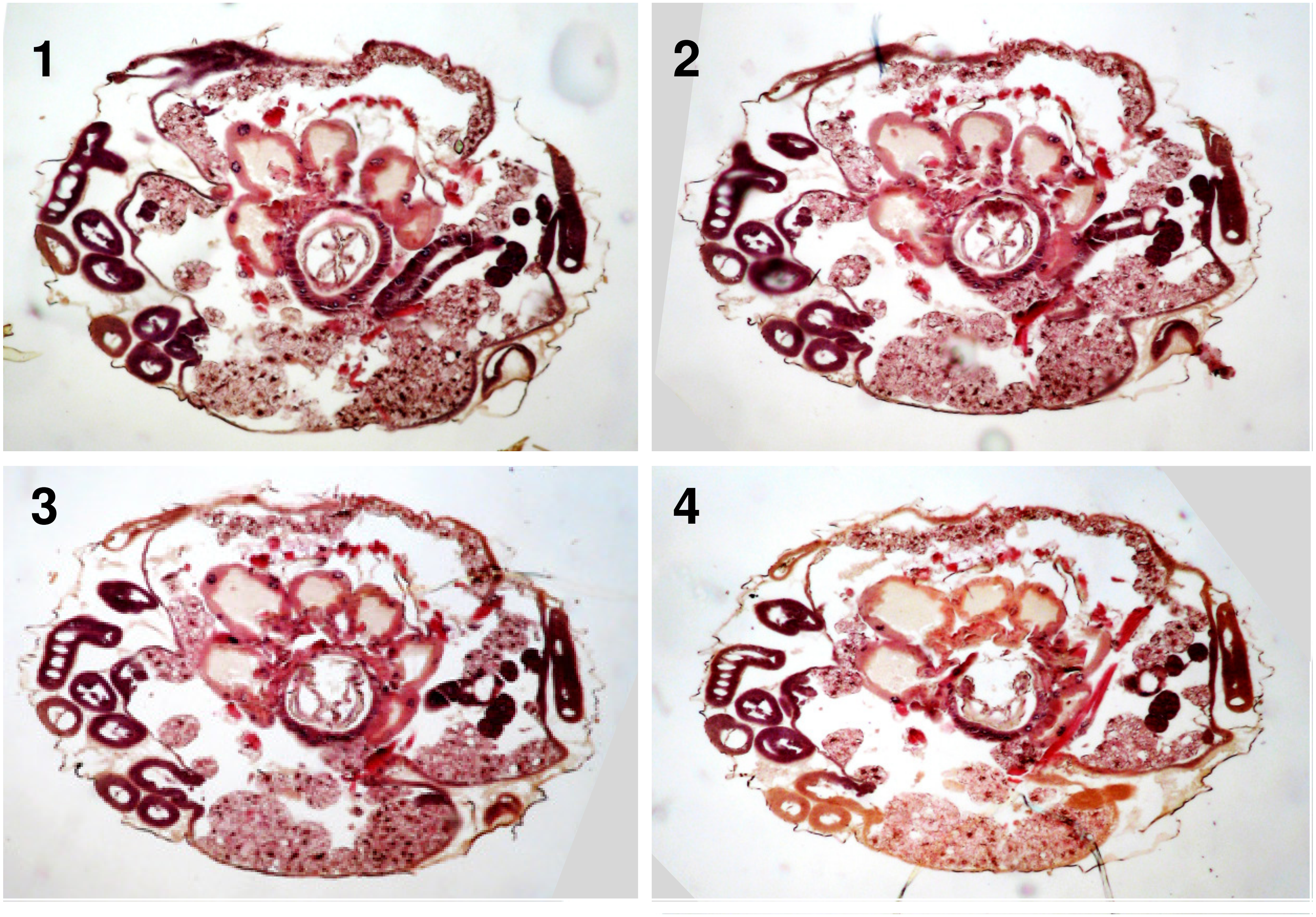

First Novaluron L4I (continued)

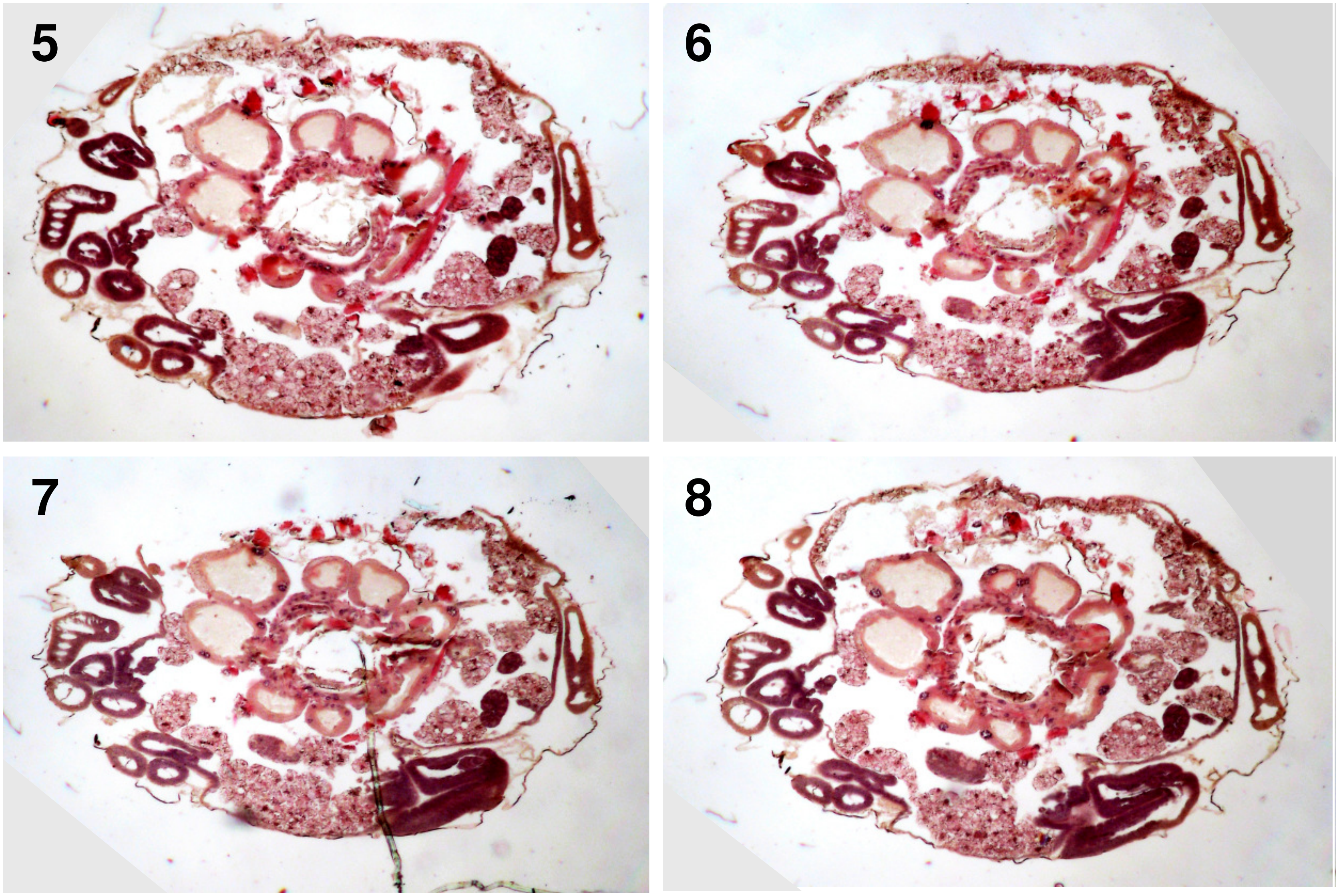

First Novaluron L4I (continued)

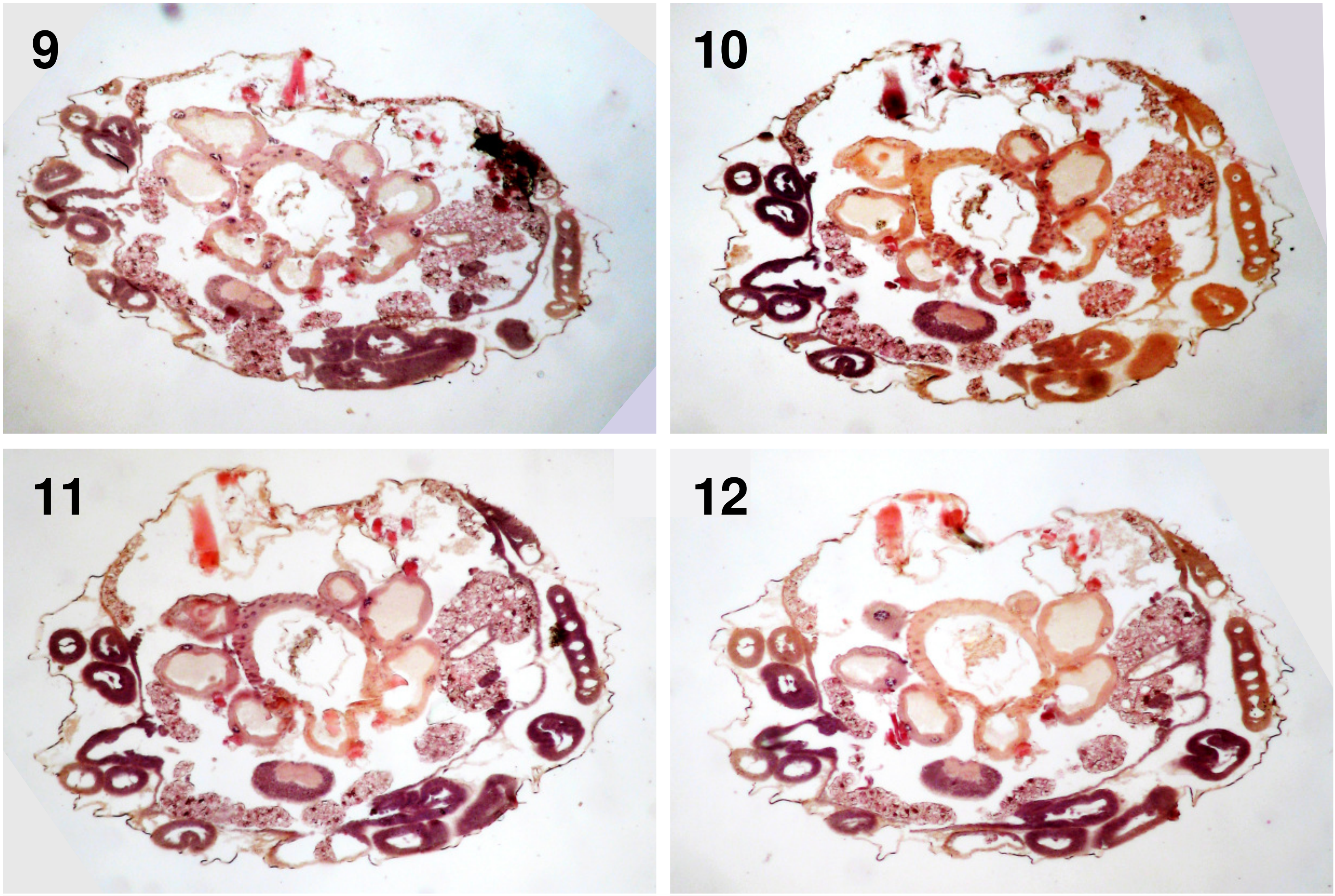

Second Novaluron L4I

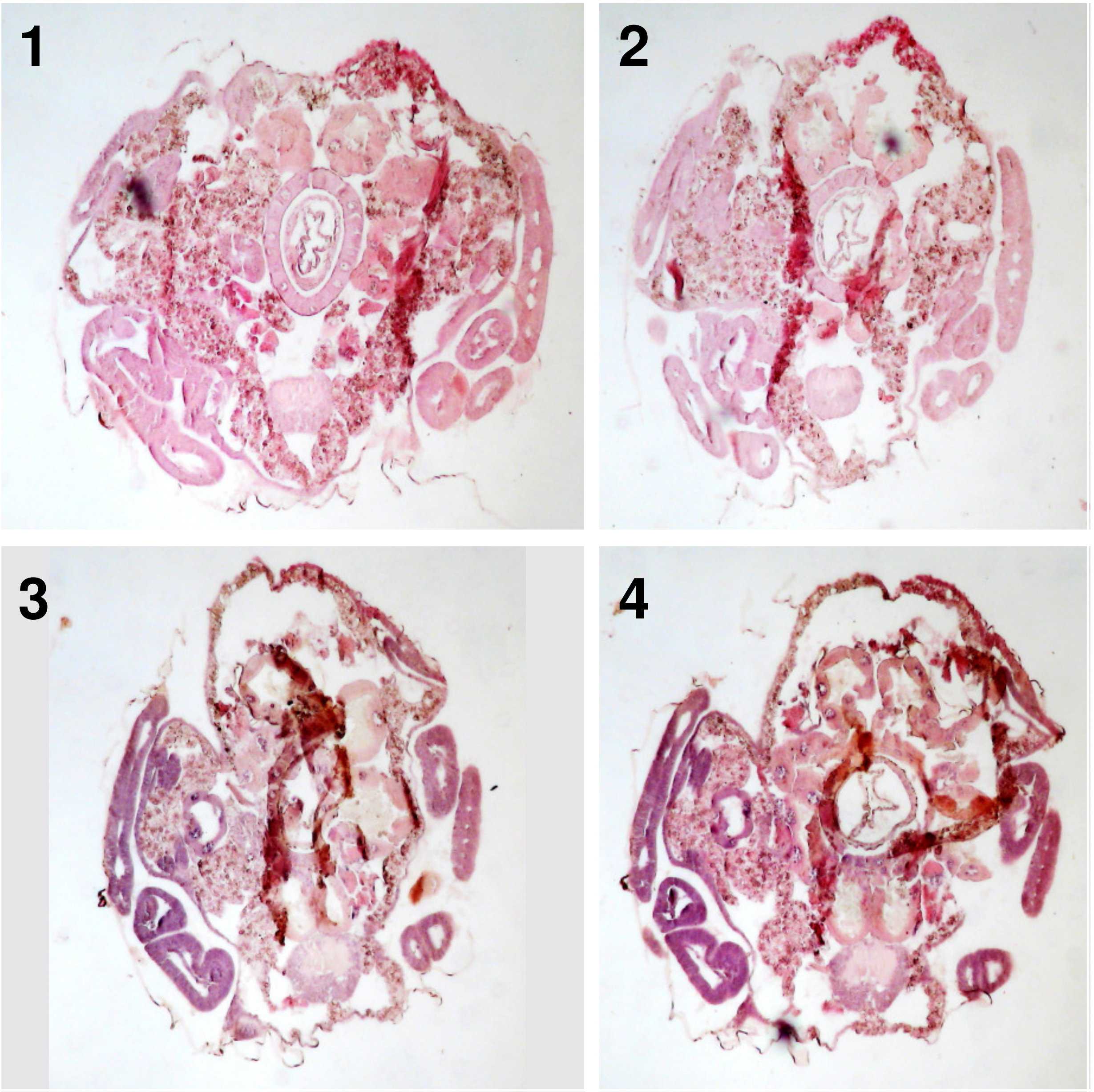

Second Novaluron L4I (continued

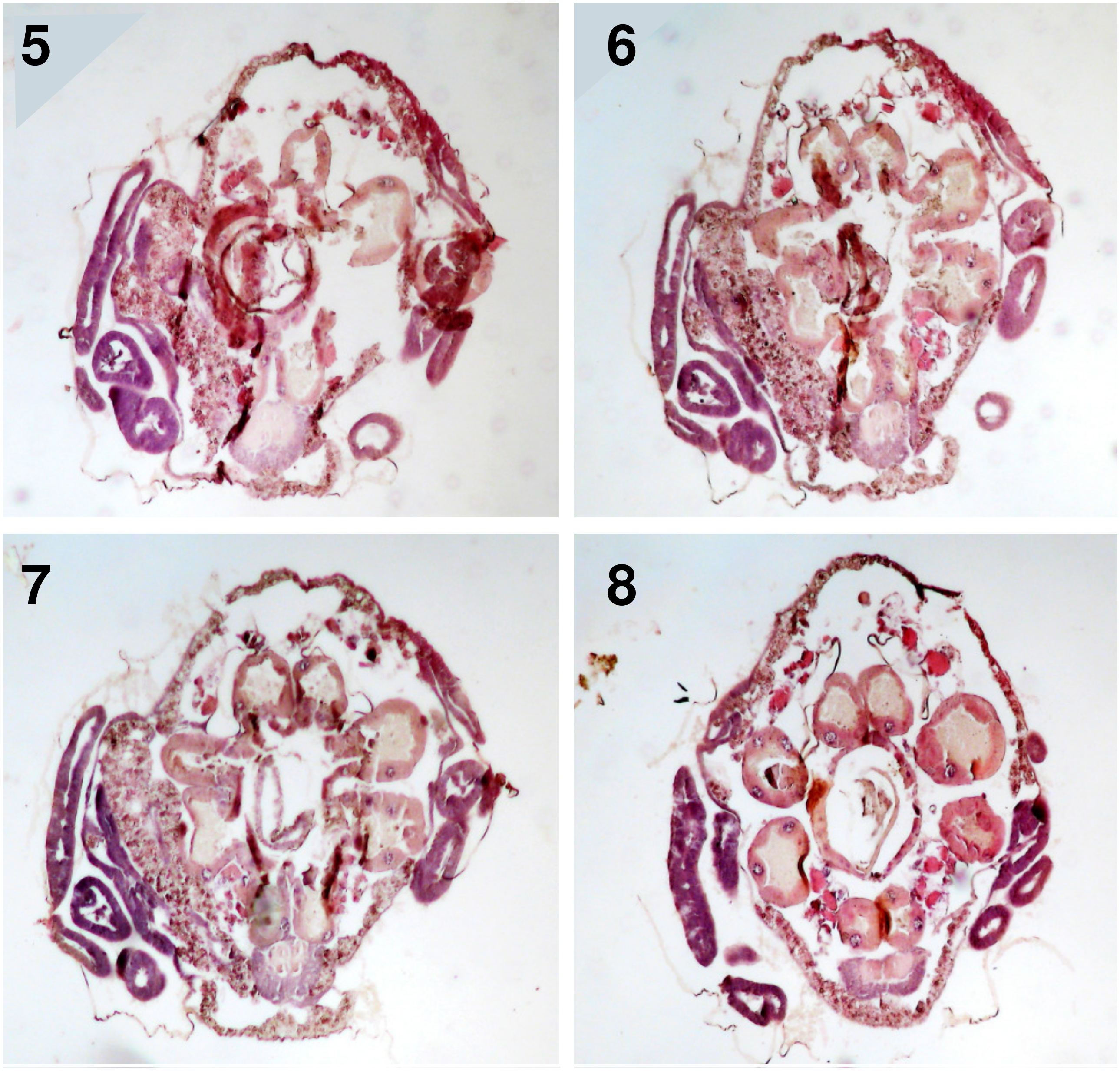

Third Novaluron L4I

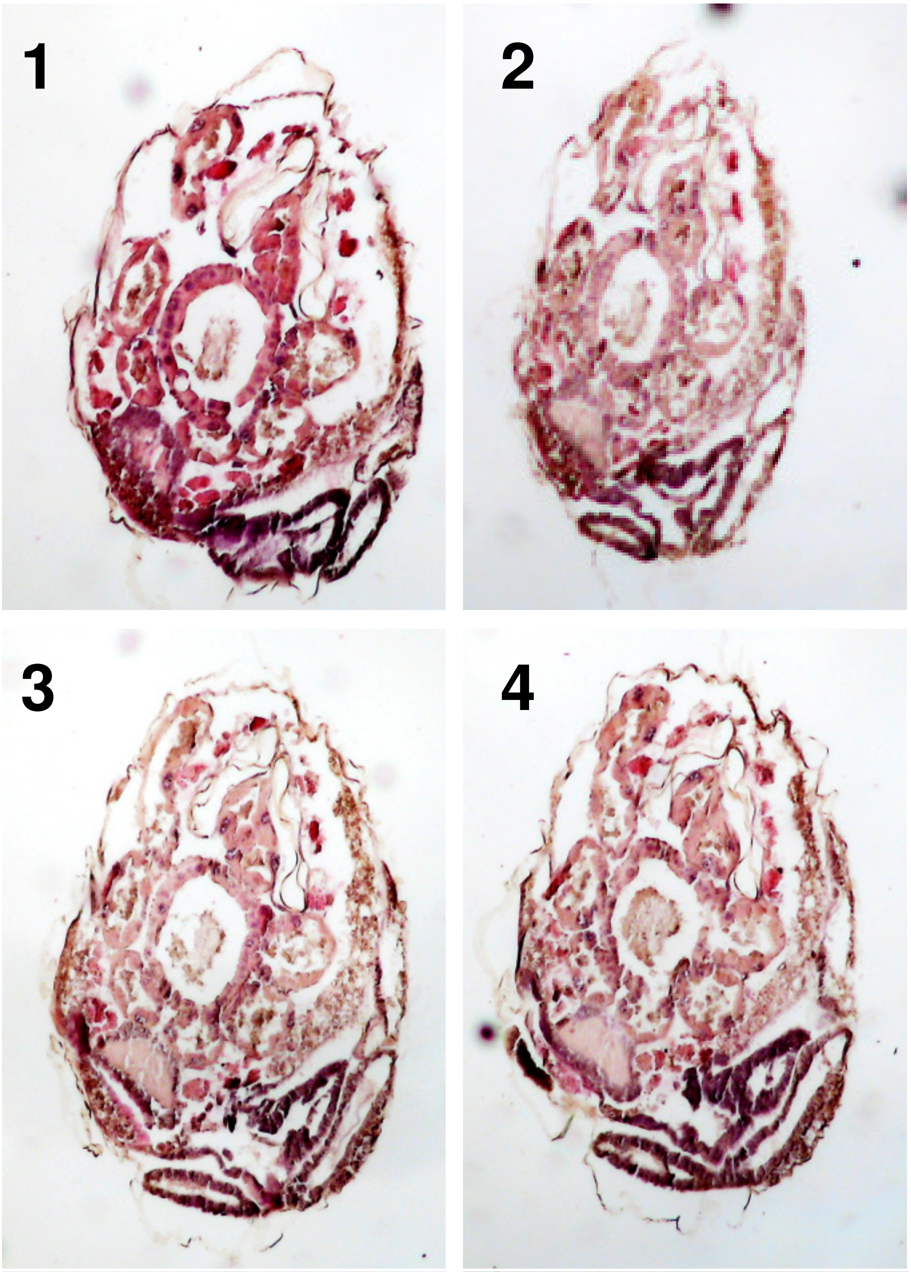

Supplement: Figure S2 — Histological cross sections of control and novaluron-treated Ae. aegypti larvae at the mesothorax region. The following slides present 45 histological cross sections of 4 control and 3 novaluron-treated (EI99) Ae. aegypti larvae at the mesothorax region. In all sections ventral side is down (i.e. the ventral thoracic ganglion is always at the bottom). Numbers in the panels denote the sequence of cross sections. For further details, please check Methods, section 7 and Figure 5 of the main text. (PDF) [file pone.0030363.s002.pdf]
